# Spinocerebellar ataxia type 31 (SCA31)

**DOI:** 10.1038/s10038-022-01091-4

**Published:** 2022-11-01

**Authors:** Kinya Ishikawa

**Affiliations:** 1 The Center for Personalized Medicine for Healthy Aging, Tokyo Medical and Dental University, Bunkyo-ku, Tokyo Japan; 2 Department of Neurology and Neurological Science, Graduate School of Medical and Dental Sciences, Tokyo Medical and Dental University, Bunkyo-ku, Tokyo Japan

**Keywords:** Microsatellite instability, Spinocerebellar ataxia

## Abstract

Spinocerebellar ataxia type 31 (SCA31) is one of the most common forms of autosomal-dominant cerebellar ataxia in Japan. SCA31 has a strong founder effect, which is consistent with the fact that this disease is basically absent in other ethnicities. After searching the entire founder region of a 2-megabase (Mb), we finally identified a 2.5 to 3.8 kb-long complex penta-nucleotide repeat containing (TGGAA)_n_, (TAGAA)_n_, (TAAAA)_n_ and (TAAAATAGAA)_n_ as the only genetic change segregating SCA31 individuals from normal people. Furthermore, (TGGAA)_n_ was isolated as the only repeat explaining the pathogenesis because other repeats were encountered in control Japanese. From the genomic point of view, the complex penta-nucleotide repeat lies in an intronic segment shared by two genes, *BEAN1* (brain expressed, associated with Nedd4) and *TK2* (thymidine kinase 2) transcribed in mutually opposite directions. While *TK2* is ubiquitously expressed, *BEAN1* is transcribed only in the brain. Thus, the complex repeat is bi-directionally transcribed exclusively in the brain, as two independent non-coding repeats. Furthermore, the complex repeat containing (UGGAA)_n_ was found to form abnormal RNA structures, called RNA foci, in cerebellar Purkinje cell nuclei of SCA31 patients’ brains. Subsequent investigation by over-expressing (UGGAA)_n_ in *Drosophila* revealed that the RNA containing (UGGAA)_n_ exerts toxicity in a length- and expression level-dependent manner, whereas its toxicity could be dampened by (UGGAA)_n_-binding proteins, TDP-43, FUS and hnRNP A2/B1. It seems rational to formulate a treatment strategy through enhancing the role of RNA-binding proteins against (UGGAA)_n_-toxicity in SCA31.

## Identification of spinocerebellar ataxia type 31 (SCA31)

**Fig. 1:**
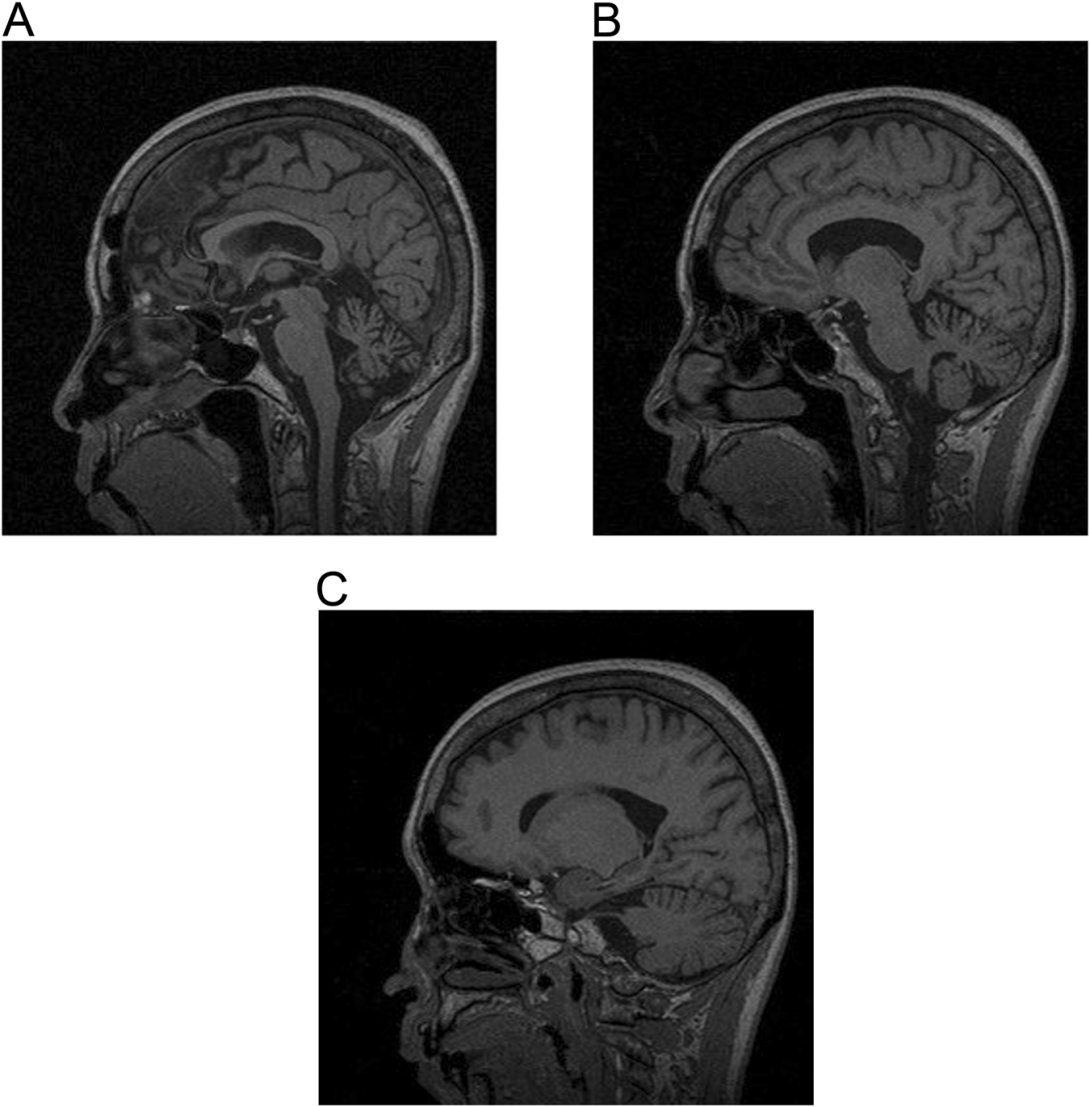
T1-weighted magnetic resonance imaging of an SCA31 patient (female, 65 years old) sectioned in the sagittal pane (**A**: near midline, **B**: paramedian, **C**: lateral sections). At a near midline plane, the culmen shows prominent atrophy and the declive, folium, tuber vermis also appear atrophic (**A**). At the lateral planes (**B**, **C**), the cerebellar atrophy is obvious only the lobulus quadrangularis

When we mapped the first group of families with pure cerebellar syndrome to human chromosome 19p, that later confirmed to be SCA6 [1], we found that there are a distinct group of families with SCA. This was the first description of SCA31 under the name of non-SCA6. Clinical features of SCA31 were summarized as, late-onset (average around 60 years old), slowly progressive ataxia. Extra-cerebellar features such as pyramidal tract signs, neuropathy or gaze limitations were not detected. In accord with this, magnetic resonance imaging showed cerebellar atrophy without brainstem involvement (Fig. 1).

We embarked a linkage analysis for these families, and three years later, we found their locus to a long arm of chromosome 16 [2]. Subsequent effort led us to finely map these families to chromosome 16q22.1 [3]. With a gradual accumulation of such families, we started to notice that affected individuals across different families share rare variants for a 2-megabase chromosomal region of 16q22.1. With a combination of these rare variants, we identified a haplotype that is not seen in control population. Therefore, SCA31 was considered to have a strong founder effect. For example, a single-nucleotide C-to-T change in the 5′ untranslated region of a gene, *PLEKHG4* for a protein with a spectrin repeat and Rho guanine-nucleotide exchange-factor domain, also called puratrophin-1 [4], appeared to be segregated with the disease. However, subsequent identification of two affected subjects from different families without this single nucleotide exchange [5, 6], suggested that the change in *PLEKHG4* was a polymorphism in a strong linkage disequilibrium (LD). The presence of strong founder effect made us to collect many families as much as we could, in order to search for a rare recombination within the locus. Thanks to many neurologists all over the country sending us their DNA samples. Continuing such efforts, we finally reached to seize a 900-kb critical region limited by two recombination events, first one as the C-to-T change in *PLEKHG4* and the other end at rs11640843 [6]: all the affected individuals across different families had identical alleles within this 900-kb region, while alleles became discordant outside the 900-kb region. We thought that the cause of SCA31 must lie somewhere within this 900-kb.

## Discovering SCA31 causative gene

In order to isolate the cause of SCA31, we had to check all the polymorphic markers that existed in the 900-kb critical region one by one. To do this, we took three different approaches: (1) Southern blot analysis for genetic rearrangement, (2) constructing BAC (bacterial artificial chromosome) tiling-path contig for the entire region followed by shot-gun sequencing for identifying smaller genetic changes, and (3) PCR-based Sanger sequencing of the entire 900-kb region. We embarked all the three approaches independently, and finally coincided with one genetic changes which all the affected members across different families harbored a 2.5 to 3.8 kb-long sequence not listed in public database [7]. Therefore, we thought this was an insertional mutation.

Cloning and sequencing of this aberrant sequence disclosed that this was a complex penta-nucleotide repeat containing (TGGAA)_n_, (TAGAA)_n_, (TAAAA)_n_ and (TAAAATAGAA)_n_ [7] (Table 1). In controls, a short, polymorphic (TAAAA)_8–20_ was seen. As we initially did not understand the implication of this change, we examined the relation between the length of this complex repeat and their age-of-onset for all available samples. Although the association was initially unclear, continuing this search by increasing new patients led us to find a weak inverse correlation between the two factors, supporting our discovery that the complex penta-nucleotide repeat containing (TGGAA)_n_, (TAGAA)_n_, (TAAAA)_n_ and (TAAAATAGAA)_n_ is indeed the mutation [7]. In addition, this insertion was not seen in a large set of control chromosomes: the vast majority (99.7%) of Japanese had a short TAAAA repeat of only 8 to 20 repeats. The only exception to this was noticed in three out of 1500 normal Japanese chromosomes: the three were, one chromosome with a long pure stretch of (TAAAA)_n_, and the other two chromosomes were complex repeats with (TAAAA)_n_, (TAGAA)_n_ and (TAAAATAGAA)_n_. Thus, (TGGAA)_n_ was never observed in controls. From these observations, we concluded that (TGGAA)_n_ was the only repeat segregating with the phenotype, suggesting its importance in pathogenesis. The presence of TGGAA repeat in SCA31 patients was also confirmed independently by other researchers on different set of families [8].

**Table 1:** The canonical repeat in the SCA31 locus is a short TAAAA penta-nucleotide repeat, usually of 8–20 repeats

	Repeat configurations	Allele frequency(%)	Pathogenic?
Japanese	(TAAAA)_8~20_	99.6^a^	No
	(TAGAA)_n_(TAAAATAGAA)_n_ expansion	0.24^a^	No
	(TGGAA)_n_(TAGAA)_n_(TAAAATAGAA)_n_ expansion	0.003^b^	Yes
European Caucasians^c^	(TAAAA)_8~20_	94.5	No
	(TACAA)_n_	5.5	No
	(TAACA)_n_		
	(TGAAA)_n_		
	(GAAAA)_n_		

However, a long, complex repeat consisting of (TAAAA)_n_(TAGAA)_n_(TAAAATAGAA)_n_ are rarely seen in Japanese. In SCA31 patients, (TGGAA)_n_ in conjunction with (TAAAA)_n_(TAGAA)_n_(TAAAATAGAA)_n_ is seen. Assuming that the disease frequency of SCA31 is around 0.003% of general Japanese population, the frequency of (TAAAA)_n_(TAGAA)_n_(TAAAATAGAA)_n_ is much higher than that of SCA31 founder chromosome.

In Caucasian population, different repeats such as (TACAA)_n_, (GAAAA)_n_, (TGAAA)_n_, and (TAACA)_n_ are seen. The frequency of these repeats are very high (5.5% of general population).

^a^Numbers came from actual data on >1000 chromosomes

^b^Calculated by assuming the disease frequency of SCA31 as 0.003% of general population, at the very most.

^c^Numebrs came from actual data on >1400 healthy and ataxia chromosomes

## The penta-nucleotide repeat responsible for SCA31 is transcribed in two mutually opposite directions

Public databases suggested that the complex penta-nucleotide repeat containing (TGGAA)_n_ lay in a region between the two genes, *BEAN1* (brain expressed, associated with Nedd4) and *TK2* (thymidine kinase 2). As databases at that time suggested that two genes were transcribed in mutually opposite directions, and their genomic structure did not contain the complex penta-nucleotide repeat, we suspected that there could be additional exons downstream for both genes. In addition, it was suggested that *BEAN1* drives brain-specific expression, while *TK2* is expressed in all human tissues. We, therefore, underwent extensive 3′-RACE experiments for both genes on brain-extracted complimentary DNA (cDNA), and found that they indeed had multiple downstream exons that had not been deposited in databases. This implied that the 2.5 to 3.8 kb-long insertion is in an intronic region shared by the two genes, *BEAN1* and *TK2* [7]. Furthermore, it was assumed that the TGGAA repeat should be transcribed as UGGAA repeat and UUCCA repeat as independent RNA repeats in SCA31 brains.

## The implication of founder effect in SCA31

As described, the SCA31 shows a strong founder effect. While SCA31 is a common ataxia in Japan, this disease has been reported from only a few countries such as Korea [9], Taiwan [10], and China [11, 12]. SCA31 was found in Brazilian SCA patients [13]. However, all these Brazilian patients were descendants of Japanese immigrants [13]. In addition, SCA31 in these four countries are extremely rare. In accord with this notion, SCA31 with (TGGAA)_n_ was never found in the Caucasian SCA families (*n* = 320) in French and German cohorts nor in the 588 healthy control subjects [14]. From these observations, it is highly likely that SCA31 give rise from the founder chromosome where (TAAAA)_n_, (TAGAA)_n_, and (TAAAATAGAA)_n_ are present.

Apart from this notion, we also found the fact that nearly 5.5% of the tested Caucasian cohorts harbored penta-nucleotide repeat expansions different from Japanese repeats. This raised an intriguing question why the SCA31 penta-nucleotide repeat locus is highly unstable depending on the ethnic backgrounds. The basic repeat is (TAAAA)_n_ regardless of the ethnicity. In Caucasians, different repeats such as (TACAA)_n_, (GAAAA)_n_, (TGAAA)_n_, and (TAACA)_n_ are frequently seen (Table 1). The most common (TACAA)_n_ sometimes showed expansion up to 6.5 kb. Even in such individuals, no obvious clinical manifestations are seen. In addition, these Caucasian repeats were all pure repeats, while the SCA31 insertion consisted of three different repeats (TGGAA)_n_, (TAGAA)_n_, and (TAAAA)_n_. It seems possible that all the Caucasian repeats and the Japanese normal repeat (TAGAA)_n_, all arose through a single nucleotide transition. For example, (TACAA)_n_ could arise from a A-to-C transition of the second A in the (TAAAA). If this view is correct, the (TGGAA)_n_ could be regarded as the consequence of two transitions of A-to-G. If this is the case, some unknown genetic factor that predisposes to transitions may be present in SCA31 genome. Furthermore, such factors may also be present in other repeat expansion disorders.

## Lessons learned from cultured cells and *Drosophila* model systems

As described, (TGGAA)_n_ is expressed in both directions: *TK2* gene drives (UUCCA)_n_ in virtually ubiquitous tissues, while *BEAN1* gene is expressed exclusively in the brain. As patients with SCA31 do not show any clear manifestation outside the brain [15, 16], we thought that *BEAN1* gene expression fitted better than *TK2* for explaining the pathogenesis. Therefore, we tested whether (UGGAA)_n_ or (UAGAAUAAAA)_n_, the *BEAN1*-derived penta-nucleotide RNA repeats, in human cerebellum. In situ hybridization using a locked nucleic acid (LNA)-oligonucleotide (TTCCA)_5_ probe, Yusuke Niimi and our colleagues identified RNA foci within SCA31 Purkinje cells’ nuclei [17]. Similar RNA foci were also detected by probes against (UAGAAUAAAA)_n_^21^. We also tested whether (UGGAA)_n_ is toxic in cultured cells by creating transient and stable expression cell systems. We found that cell toxicity and formation of RNA foci were both consistently observed upon expression of (UGGAA)_n_ than by expressing (UAGAAUAAAA)_n_. These observations led us to conclude that (UGGAA)_n_ could be toxic in cells.

Collaborating with Professor Yoshitaka Nagai, Taro Ishiguro and our colleagues created *Drosophila* models expressing an expanded (UGGAA)_n_ repeat RNA [18]. We convinced that the expanded (UGGAA)_n_ are toxic in *Drosophila* as well. In this model, the length of penta-nucleotide repeat had been contracted to 80–100 repeat (UGGAA)_n_ compared to human SCA31 genome. Nevertheless, abundant RNA foci and remarkable eye degeneration were seen: some of the strongest lines were lethal, and some other lines with dramatic eye degenerations had a strong transgene expressions. On the other hand, lines with mild phenotypes had a low (UGGAA)_n_ expression, in which the number of RNA foci was also small. The control repeat and a short UGGAA_22_ RNA, the latter happened to be generated by spontaneous contraction of the TGGAA repeats, had no significant effect.

Considering that one of the pathogeneses of noncoding repeat expansion disorders is mediated by RNA-binding proteins by binding to noncoding repeat responsible for human diseases [19], we thought that there could be (UGGAA)_n_ binding proteins that may mediate some sort of disease mechanisms. Therefore, Sato N. and our colleagues screened for potential (UGGAA)_n_-binding proteins by in vitro RNA pull-down assay using nuclear fraction of PC12 cells, and found that TDP-43 (TAR DNA-binding protein, 43 kilodalton), FUS, and hnRNPs may bind to (UGGAA)_n_. After confirming that TDP-43 would bind to (UGGAA)_n_ in western blot [18], we crossed (UGGAA)_n_ expressing flies with a line that expresses human TDP-43, and found that the phenotype and RNA foci brought by (UGGAA)_n_ were both alleviated. Similar effects were also seen for other (UGGAA)_n_ binding proteins, FUS and hnRNPA2/B1. When the (UGGAA)_n_ expressing flies were crossed with the fly, in which the endogenous protein homologous to TDP-43 was deleted, the (UGGAA)_n_ toxicity was further enhanced. Further details of our findings could be seen in reference [18]. As these RNA binding proteins were thought to fix abnormal RNA structure of (UGGAA)_n_, we considered these proteins act as RNA chaperone.

From these and other related findings, we built a new idea that the RNA toxicity initiated by (UGGAA)_n_-toxicity and its counteracting effect by the (UGGAA)_n_-binding proteins as RNA chaperones are maintained in a well-balanced state when healthy, but once it tilts towards the RNA toxicity due to overexpression of (UGGAA)_n_, SCA31 emerges.

## Conclusions

If our hypothesis is true for SCA31 human brains, overexpression of RNA binding proteins to an appropriate level may be rational, as we saw in *Drosophila* models.

However, the number of (UGGAA)_n_-containing RNA foci is low. Therefore, it seems that there are many other hidden factors behind SCA31 pathogenesis. Further studies are needed to discover mechanisms that (TGGAA)_n_ in SCA31 patients’ genome leads to neurodegeneration.
